# Process Correlation Analysis Model for Process Improvement Identification

**DOI:** 10.1155/2014/104072

**Published:** 2014-03-24

**Authors:** Su-jin Choi, Dae-Kyoo Kim, Sooyong Park

**Affiliations:** ^1^Sogang University, Seoul, Republic of Korea; ^2^Oakland University, Rochester' MI 48309, USA

## Abstract

Software process improvement aims at improving the development process of software systems. It is initiated by process assessment identifying strengths and weaknesses and based on the findings, improvement plans are developed. In general, a process reference model (e.g., CMMI) is used throughout the process of software process improvement as the base. CMMI defines a set of process areas involved in software development and what to be carried out in process areas in terms of goals and practices. Process areas and their elements (goals and practices) are often correlated due to the iterative nature of software development process. However, in the current practice, correlations of process elements are often overlooked in the development of an improvement plan, which diminishes the efficiency of the plan. This is mainly attributed to significant efforts and the lack of required expertise. In this paper, we present a process correlation analysis model that helps identify correlations of process elements from the results of process assessment. This model is defined based on CMMI and empirical data of improvement practices. We evaluate the model using industrial data.

## 1. Introduction

Software process improvement (SPI) is carried out to improve the efficiency of software development process by analyzing involved practices and their relations in consideration of available resources in an organization. SPI is initiated by assessing the current process to identify strengths and weaknesses. Based on findings, improvement plans are developed to reinforce strengths and improve weaknesses.

In general, a reference model is used throughout SPI as the base. A widely used model is Capability Maturity Model Integration (CMMI) [1] which has shown its impact on product quality, development cost, and time-to-market across the industry [2–4]. CMMI provides a structured process assessment framework defined upon a set of process areas (PAs). 22 PAs (e.g., project planning and requirement definition) are defined each describing specific goals to be achieved and specific practices to be carried out. PAs and their components (goals and practices) are highly correlated, which reflects the iterative nature of software development process [5]. These correlations capture dependencies among PAs and components which should be considered in improvement planning [6, 7].

However, the current practice focuses only on individual PAs and often overlooks correlations of PAs, which diminishes the efficiency of improvement plans. This is mainly attributed to significant efforts and the lack of required expertise. There exist a few studies on identifying correlations of improvement items (e.g., CMMI practices and improvement agendas) and improvement planning [7–10]. The existing work, however, largely relies on analyst's expertise and manual work which make it difficult for the less experienced to practice. Some works (e.g., [7, 8, 10]) propose manual approaches for reviewing and relating process items and some others (e.g., [9]) present an expert-friendly template for describing improvement agendas.

In this paper, we present a process correlation analysis model for identifying correlations of PAs, goals, and practices based on CMMI and empirical data collected from SPI projects where the analysis of findings' correlations have been done. CMMI is used to infer correlations of PAs and their components and the inferred correlations are affirmed and complemented using empirical data. The model takes as input the findings of process assessment and identifies their correlations by analyzing the findings to corresponding practices correlated in CMMI and empirical data. The model then produces a graph representing correlations of findings. We evaluate the model in terms of precision, recall, F-measure, and accuracy using five different industrial SPI projects. We also demonstrate tool support for the presented model. In our previous work, we presented ReMo, a model for developing improvement recommendations which use manual correlation analysis. This work complements the previous work by providing a systematic way of identifying process correlations.

The paper is structured as follows.  Section 2 gives an overview of related work, Section 3 details the proposed process correlation analysis model and its supporting tool, Section 4 presents the evaluation results using industrial data, and Section 5 concludes the paper with future work.

## 2. Related Work

There is some work on identifying improvements using a certain type of relationships. Gorscheck and Wohlin [8] propose* DAIIPS*, a method for packaging improvement issues by prioritizing them based on analysis of their dependencies. This method is designed for small organizations where resources for addressing improvement issues are limited. The degree of dependency between improvement issues is decided by vote in a workshop. That is, the decision on dependency is greatly influenced by the level of participants' expertise. Moreover, they do not provide criteria for the qualification of workshop attendees and guidelines for making dependency decisions. Such a manual process is time consuming and requires significant efforts.

Calvo-Manzano Villalón et al. [9] present an improvement specification template called* Action Package* for describing organizational, technical, and management aspects of process improvements. The template consists of twelve items including policy, training, and metrics. However, the template is designed for experts, providing little details as to how the items should be filled out. This makes it difficult for the less experienced to use the template.

Sun and Liu [10] present a CMMI-based method for relating improvement issues to CMMI practices using the Quality Function Deployment technique [11]. Similar to Gorscheck and Wolin's work, they prioritize improvement issues by the number of relations to practices. The higher the number of relations, the higher the priority. However, the method does not describe how identified relations may be used.

Chen et al. [7] present a practice dependency model for CMMI using six process areas at level 2. Dependencies between practices are identified via the flow of work products between practices based on a textual analysis of the CMMI specification. Their work is similar to our work in that our work also uses CMMI. However, the specification of CMMI involves many ambiguities, which limits the extent to which CMMI alone may provide information about correlations. To address this, we make use of empirical field data from industry in addition to CMMI.

## 3. Correlation Analysis Model

**Figure 1:**
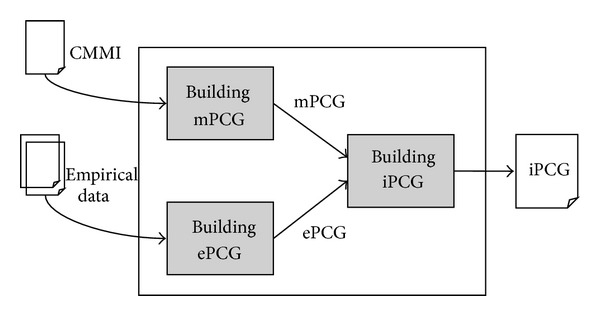
Process correlation model.

In this section, we describe a process correlation analysis model that identifies correlations from a given assessment finding set. The model is built upon CMMI and empirical field data. CMMI is used as a base for identifying an initial set of common correlations of practices from its descriptions on PAs and their components (e.g., goals and practices). Identified correlations are represented as a graph. The graph is referred to as* mPCG* which denotes a process correlation graph (PCG) for the considered model (CMMI). However, the description in CMMI involves ambiguities and inconsistencies due to its general nature. To address this, we use empirical data collected from various SPI projects in addition to CMMI. Correlations are identified from empirical data and represented as a graph referred to as* ePCG*.* mPCG* and* ePCG* are then combined to produce an integrated graph* iPCG*.  Figure 1 illustrates the process of the model. Henceforth, we use terms correlation and relation interchangeably.

### 3.1. Building* mPCG*

We use CMMI as the base for identifying practice correlations. CMMI describes a PA in terms of *Goals*, *Practices*, and *Subpractices*. Goals may be generic or specific. A specific goal is achieved by a set of specific practices producing certain work products. A specific practice may consist of a set of subpractices. The specific goals, practices, and subpractices of a PA may reference the components of another PA. Generic goals are shared by PAs and, thus, there is no reference for generic goals.

**Figure 2:**
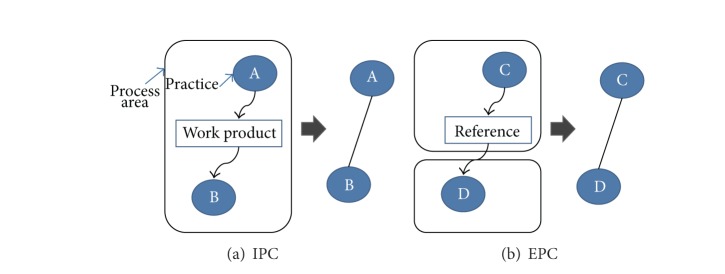
Identifying practice correlations in CMMI.

Given the PA descriptions in CMMI, we look for information about internal practice correlations (IPCs) and external practice correlations (EPCs). IPCs exist within the same process area while EPCs cut across process areas. To identify IPCs, we make use of the description of practices and subpractices and their related work products. Specifically, we focus on relationships of input and output work products among practices. Two internal practices are considered as internally correlated if one practice has output work products that are used as input work products of the other.  Figure 2(a) shows an IPC. EPCs are identified based on the description of related process areas and external references. Two external practices are considered as externally correlated if one practice refers to the other, which is shown in Figure 2(b).

**Figure 3:**
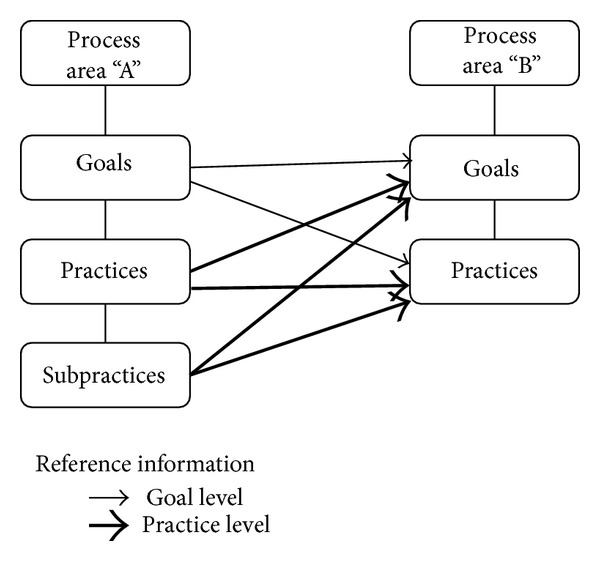
Structure of process areas in CMMI.

Goals, practices, and subpractices are defined at different levels as shown in Figure 3. The goal level is the highest followed by the practice level and then the subpractice level. We consider two components (e.g., goals) as correlated if they reference each other. A component referencing another component at a lower level is considered as related to that specific component only. On the other hand, a component referencing another component at a higher level is considered as related to all the subcomponents of the component. Two PAs are considered as correlated if a component of one PA refers to a component of the other or one PA refers to the other PA in its description.

Table 1 shows examples of PAs and a subset of their components defined in CMMI. The table describes three PAs—*Requirement Management (REQM)*,* Project Monitoring and Control (PMC)*, and* Project Planning (PP)*.* REQM* has one specific goal (*SG 1*) with two specific practices (*SP 1.1* and* SP 1.2*). Other PAs can be explained similarly. A component may be accompanied with reference information. For instance,* SG 1* in* REQM* has the following reference description: “*Refer to the *Project Monitoring and Control* process area for more information about *managing corrective action to closure.” From this description, one can obviously infer a relation of* REQM SG 1* to the* SG 2* goal of* PMC*, which is an example of a goal-level reference. Similarly, from the reference description of* REQM SP 1.2*, it can be easily inferred that* REQM SP 1.2* is related to the* SP 1.2* practice in* PMC*.

**Figure 4:**
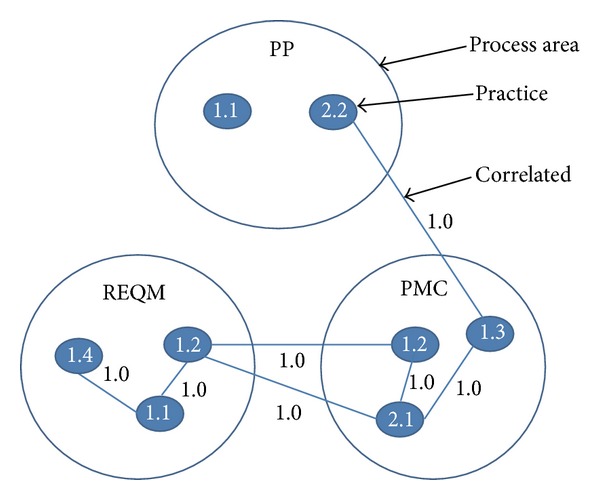
mPCG example.

Based on elicited correlations of PAs and their components, a mPCG is built. A mPCG is a nondirected graph capturing correlations of practices where a node represents a practice and an edge represents a correlation. An edge between nodes *p*_*m*_ and *p*_*n*_ has a weight denoted by *W*_*m*(*p*_*m*_,*p*_*n*_)_. All edges in a mPCG have their weight 1 denoting the existence of a correlation. Correlations may be found within the same PA or across PAs.  Figure 4 shows an example of a mPCG.

Note that CMMI descriptions are not always explicit. For instance, subpractices are described mostly about output work products while having little description on input products. Goal-level references across PAs are described abstractly providing little information on how they influence related practices. CMMI descriptions on process areas also involve ambiguities.

### 3.2. Building* ePCG*

To address the above, we make use of empirical data collected from a set of industrial SPI projects in addition to CMMI. The data is postproject data including findings and their correlations are already analyzed and used in completed projects. Postproject findings are often tagged with relevant CMMI practices. Accordingly, we assume that they are all tagged in this work.

**Figure 5:**
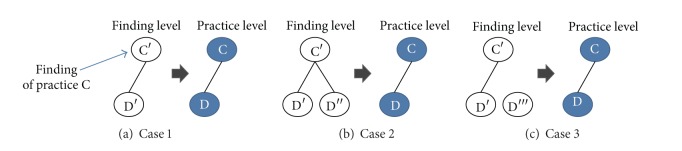
Correspondence between findings and practices.

Each project is analyzed to identify correlated practices which are captured in a PCG. PCGs of all the considered projects are combined to produce an ePCG. The resulting ePCG is then merged with the mPCG of CMMI. However, unlike CMMI where correlations are described for practices, empirical data are described for findings which are implementations of practices. This is because an SPI project is an implementation of CMMI. Given that, findings in empirical data should be abstracted to practices to align the level of abstraction of ePCGs with the mPCG of CMMI. Abstraction is carried out as follows.

 (R1)  For each finding, identify the corresponding practice in CMMI.
 (R2)  Two practices are considered as correlated if a finding of the practice is related to a finding of the other practice. This is illustrated in Figure 5(a). The same holds regardless the number of instances (findings) of a practice. Figures 5(b) and 5(c) show the cases where a practice has multiple instances with different relations. Both cases result in the same abstraction as Figure 5(a) per R3.

We demonstrate abstraction using the findings in Table 2. The table shows four findings* REQM-W-01*,* PP-W-01*,* PP-W-02*, and* PP-S-01* where* REQM-W-01* is an instance of the practice* REQM SP 1.4* in Table 1 and* PP-W-01*,* PP-W-02*, and* PP-S-01* are instances of* PP SP 1.1*. From the description of the findings,* REQM-W-01* and* PP-W-01* are found related since requirement traceability needs to be maintained for development tasks and work products defined in work breakdown structure. Similarly,* REQM-W-01* and* PP-S-01* are found related since* PP-S-01* is a strength practice of defining development tasks and work products. Given this relation, the corresponding practices* REQM SP 1.4* and* PP SP 1.1* to these findings are considered also as related. On the other hand,* REQM-W-01* and* PP-W-02* are found not related because COTS products are already made. Thus, requirements traceability to development tasks and work products is not necessary. However, their corresponding practices are considered as related as they have already been so for other pairs.

PCGs of SPI projects are merged by combining nodes and edges to create an ePCG for the considered project set. Each edge is weighted by the number of projects having the edge identified in their PCG. Let |*P*_*p*1,*p*2_| be the number of the projects whose PCG has practices *p*1 and *p*2 correlated and $\left|P_{\hat{p1,p2}}\right|$ the number of the projects whose PCG has *p*1 and *p*2 identified as correlated. Then, the weight *W*_*e*(*p*_*m*_,*p*_*n*_)_ of the edge between *p*_*m*_ and *p*_*n*_ is defined as follows:

(1) $$\begin{array}{c} W_{e(p_{m},p_{n})}=\frac{\left|P_{\hat{\left(p_{m},p_{n}\right)}}\right|}{\left|P_{\left(p_{m},p_{n}\right)}\right|},\;\left(0\leq W_{e(p_{m},p_{n})}\leq 1\right). \end{array}$$

**Figure 6:**
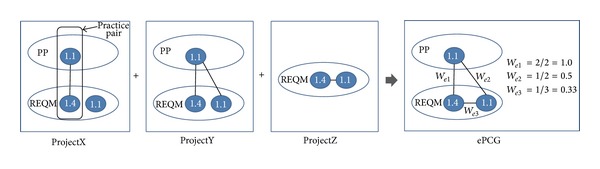
Building an ePCG.

As an example, consider Figure 6. In the figure, there are three projects* ProjetX*,* ProjectY*, and* ProjectZ* where the PCG of* ProjetX* has one related practice pair ((*PP 1.1, REQM 1.4*)), the PCG of* ProjectY* has two related pairs ((*PP 1.1, REQM 1.4*), (*PP 1.1, REQM 1.1*)), and the PCG of* ProjectZ* has one related pair ((*REQM 1.4, REQM 1.1*)). The practices are the same as those in Figure 4. PCGs of the projects are merged into an ePCG by adding all the nodes (*PP 1.1*,* REQM 1.4*,* REQM 1.1*) and their relations involved in the PCGs. The weight of the edge between* PP 1.1* and* REQM 1.1* is measured at 0.5 (1/2) as the number of the projects that involve* PP 1.1* and* REQM 1.4* is two (ProjectX, ProjectY) and the number of the projects that have* PP 1.1* and* REQM 1.1* identified as correlated is one (ProjectY). Weight for other edges can be measured similarly.

### 3.3. Building* iPCG* by Combining* mPCG* and* ePCG*

**Figure 7:**
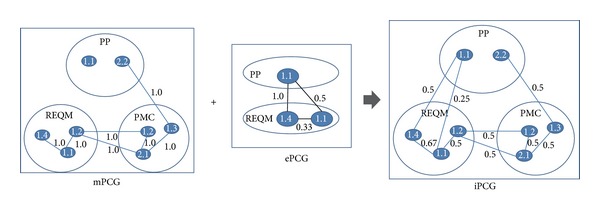
Building an iPCG.

The mPCG resulting from Section 3.1 and the ePCG from Section 3.1 are merged to produce an integrated graph iPCG.  Figure 7 shows an example which combines the mPCG in Figure 4 and the ePCG in Figure 6.

The weight *W*_*i*(*p*_*m*_,*p*_*n*_)_ of an edge between *p*_*m*_ and *p*_*n*_ in the iPCG is computed as follows:

(2) $$\begin{array}{c} W_{i(p_{m},p_{n})}=\frac{W_{m(p_{m},p_{n})}+W_{e(p_{m},p_{n})}}{2},\;\left(0\leq W_{i(p_{m},p_{n})}\leq 1\right). \end{array}$$

A threshold is set for weight and any edge having its weight lower than the threshold is considered as not related. We use 0.25 for the threshold in this work. This means that a practice pair in an iPCG is considered as correlated if it is identified in CMMI (*W*_*m*_ = 1) or in the half or more of the empirical projects (*W*_*e*_ ≥ 0.5).

**Figure 8:**
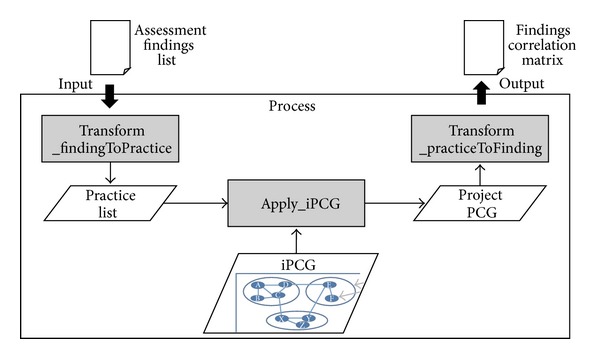
Application process of the correlation model.

Figure 8 shows the application process of the presented model. The findings identified in the process assessment of an SPI project are abstracted to practices using the abstraction rules in Section 3.2. The resulting practices are then input to the iPCG to produce a PCG of the project described in terms of practices. The practices in the PCG are concretized back to findings using the same mapping used as in abstracting the findings to practices at the beginning.

**Figure 9:**
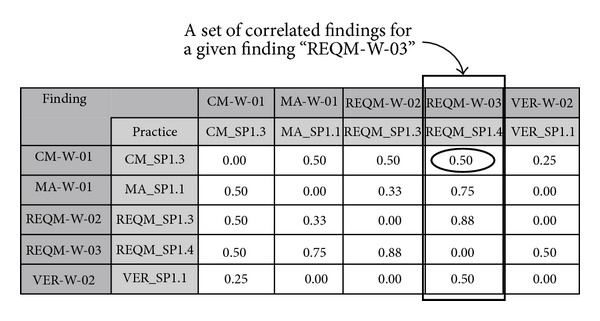
Example of resulting finding correlation.

Figure 9 shows an example of a resulting correlation matrix. The matrix is symmetric having the same set of findings in column and row and the values represent weights. In the figure, the box in bold line shows that the weight of the (*REQM-W-03, CM-W-01*) pair is measured as 0.50 which indicates that the practices in the pair are correlated in either CMMI (*W*_*m*_ = 1) or the empirical data (*W*_*e*_ = 1) used in the model. Those pairs that have zero weight are not related. The resulting matrix is suggestive. That is, the process analyst may modify the matrix at his discretion. The matrix can be also used for identifying more significant findings by prioritizing them by the number of correlations or accumulated weights (i.e., adding all the weights by column).

### 3.4. Supporting Tool

**Figure 10:**
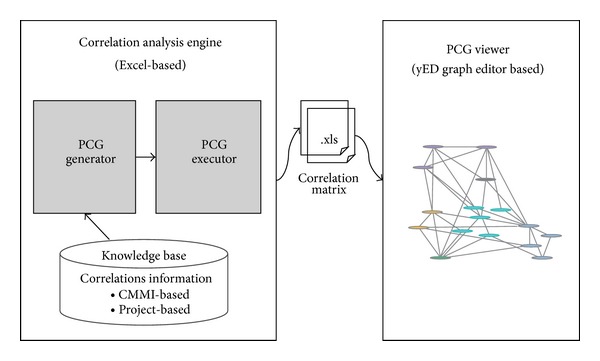
Prototype tool components.

**Figure 11:**
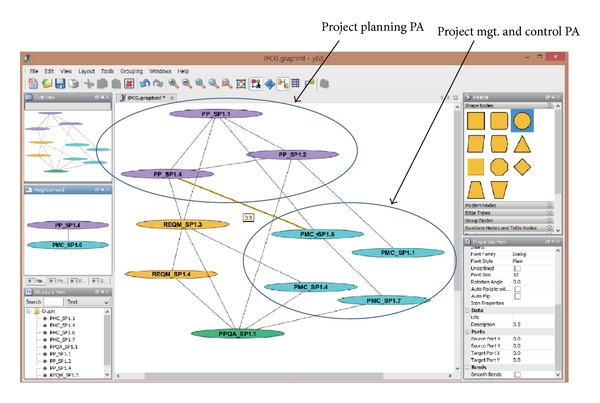
PCG viewer.

We have developed a prototype tool supporting the presented model.  Figure 10 shows the architecture of the tool. It consists of two components—a correlation analysis engine and a PCG viewer. The correlation analysis engine is Excel-based consisting of (1) a knowledge base containing CMMI-based practice correlations and project-based practice correlations, (2) a PCG generator responsible for building PCGs, and (3) a PCG executor applying an iPCG and generating the output correlations in an Excel file. The PCG viewer displays the resulting PCG using the* yED Graph Viewer*, an open source application for visualizing object connections [12]. The viewer takes an input file in various formats including the Excel files generated by the PCG executor.  Figure 11 shows a screenshot of the PCG viewer. Nodes are grouped to denote different PAs of relevance and correlation weights are displayed with mouse-over on edges.

## 4. Evaluation

We evaluate the presented model in terms of recall, precision, F-measure, and accuracy [13] by comparing the resulting correlations to manually identified correlations by experts. Recall is measured by the number of correlations produced by the model over the number of manually identified correlations. Similarly, precision is measured by the number of manually produced correlations over the number of correlations produced by the model. The accuracy is measured by the number of correctly identified correlations to the total number of practice pairs.

We use five industry SPI projects to evaluate the model each from a different company. Four projects are used for building the iPCG in the model and the remaining project is used as the target project to which the model is applied. The target project is rotated in the five projects so that the model can be applied to all the five projects. As the target project is rotated, the project used as the target project is excluded from the project set used to build the iPCG in the model. In this way, the iPCG is not biased to the target project. The five projects are all CMMI-based targeting the maturity level from 2 to 4.

Table 3 shows an overview of the five projects used in the evaluation. In the table, *CompA* and *CompB* aim at level 3, *CompC* and *CompD* aim at level 2, and *CompE* aims at level 4. Given that, it can be observed in the table that the four PAs (*requirement management*,* project planning*,* measurement and analysis*,* and configuration management process*) in *CompC* and *CompD* are from level 2 and the two additional PAs (*technical solution* and* verification process*) in *CompA*, *CompB*, and *CompE* are from level 3. The table shows that for *CompA*, 20 findings are found in six PAs and they are all related to 18 practices. 49 practice correlations are found out of total 153 practice pairs in the six PAs. Other projects can be explained similarly. The six PAs involve total 50 practices in CMMI of which 40 practices (accounting for 80%) are addressed in the five projects.

Table 4 shows measured data from the evaluation.* True Positive* denotes the number of correlations that are identified by the presented model as related and also evaluated as related by experts.* True Negative* represents the number of correlations that are identified by the model as not related and also evaluated by experts as not related.* False Positive* shows the number of the correlations that are identified by the model, but are evaluated as irrelevant by experts.* False Negative* is the number of the correlations that are evaluated as related by experts, but not identified by the presented model. The table shows* CompB* having a low true positive. This is due to the lack of thoroughness in the project which was conducted hastily with nonexperts involved.* CompD* shows a relatively higher true positive and a low false positive. This is because there are only 10 practices involved in the project. Thus, it was easier for experts to identify correlations.

Based on Table 4, precision, recall, f-measure, and accuracy of the model are measured for the five projects. Precision is measured by* True Positive*/(*True Positive* +* False Positive*) while recall is measured by* True Positive*/(*True Positive* +* False Negative*); Table 5 shows the results. The table shows that the average precision is 0.57 which implies that about 60% of the correlations identified by the presented model are also identified as correlated in the five projects. The average of recall is measured as 0.51 which means that about the half of the manually identified correlations are also identified by the model.* F*-measure, which is the harmonic mean of precision and recall, is measured at 0.54.

A higher precision implies that experts have more correlations identified manually that match with the correlations identified by the model. On the other hand, a higher recall implies that the model identified more correlations matching with manually identified correlations.* CompB* shows the lowest precision and recall. This is because there is only a small number of manually identified correlations to the total number of practice pairs (which is only 19% while the average is 46%). On the other hand,* CompD* has 73% of the total number of practice pairs identified as correlated, which contributes to its highest precision and recall. This observation was expected as precision and recall depend on the number of manually identified correlations.

Accuracy is measured by (*True Positive* +* True Negative*)/(*True Positive* +* True Negative* +* False Positive* +* False Negative*). Accuracy is measured at 0.69 in average, which means that about 70% of the correlations identified by the presented model are considered as correct in the five projects. The standard deviation of accuracy is measured at 0.07 which is relatively low compared to precision and recall. This means that the presented model is consistent to the considered projects.

## 5. Conclusion

We have presented a process correlation analysis model for identifying correlations of findings. A major advantage of the model is the use of empirical data which complements the CMMI specification being ambiguous and abstract. The evaluation of the model shows 0.51 for recall and 0.69 for accuracy which demonstrates the potential of the model. It is noteworthy to mention that the model is developed in response to the need of techniques for identifying finding correlations from the field. We shall continue to improve and evaluate the model by applying it to more case studies. As more case studies are conducted, we shall extend the evaluation to look into the efficiency aspect of the model over manual analysis.

We also plan to investigate an effective way of packaging findings based on identified correlations to build improvement plans. Using correlation information, a finding that has more correlations can be found and as such a finding can lend itself as a seed for forming a package. The package may include findings that are directly correlated by the seed. The resulting package then serves as an early version of an improvement plan and may evolve throughout a series of refinement activities.

The model can be also used in the case where empirical data is not available. In such a case, one may start analyzing correlations using only mPCG and then use the empirical data from the current project as it is completed. The data then can be used as input to build an ePCG for the next project. We expect that the model is to be more accurate as more empirical data is used.

## Figures and Tables

**Table 1:** Process area examples.

Process area	Goal	Practice	Description	Reference information
Requirement management (REQM)	^ a^SG 1		Manage requirements	**Refer to** the *Project Monitoring and Control* process area for more information about managing corrective action to closure.
		^ b^SP 1.2	Obtain commitment to requirements	**Refer to** the *Project Monitoring and Control* process area for more information about monitoring commitments.
		SP 1.4	Maintain bidirectional traceability of requirements	*Not defined *

Project planning (PP)	SG 1		Establish estimates	*Not defined *
		SP 1.1	Estimate the scope of the project	**Refer to** the *Supplier Agreement Management* process area…
	SG 2		Develop a project plan	*Not defined *
		SP 2.2	Identify project risks.	**Refer to** the *Monitor Project Risks* specific practice in the *Project Planning* process area for more information about risk monitoring activities.

Project monitoring and control (PMC)	SG 1		Monitor the project against the plan	*Not defined *
		SP 1.2	Monitor commitments	*Not defined *
		SP 1.3	Monitor project risks	**Refer to** the *Project Planning* process area for more information about identifying project risks.
	SG 2		Manage corrective action to closure	*Not defined *
		SP 2.1	Analyze issues	**Refer to** the *Establish the Budget and Schedule* specific practice in the *Project Planning* process area for more information about corrective action criteria.

^a^Specific goal, ^b^specific practice.

**Table 2:** Assessment findings and corresponding practices.

Finding ID	Finding description	Practice ID
REQM-W-01	It is difficult to trace defined requirements to development tasks and work products.	REQM SP 1.4

PP-W-01	Work break down structure are developed in high level (e.g., milestone) without detailed tasks and work product.	PP SP 1.1

PP-W-02	Product components to be purchased as COTS (commercial-off-the- shelf) are not documented.	PP SP 1.1

PP-S-01	In some projects, WBS with detail description on tasks and work products is developed with the support of Quality Assurance team.	PP SP 1.1

**Table 3:** Summary on collected data.

Company	Number of process areas^1^	Number of Findings	Number of practices^1^	Number of practice pairs	
				Total	Correlated
CompA	6	20	18	153	49
CompB	6	21	19	171	32
CompC	4	12	15	105	52
CompD	4	11	10	45	33
CompE	6	22	20	190	49

Total	6	86	40	664	215

^1^Total means number of distinct process areas or practices.

**Table 4:** Measured data.

Company	True positive	True negative	False positive	False negative
CompA	25	76	28	24
CompB	11	105	34	21
CompC	20	44	9	32
CompD	23	8	4	10
CompE	30	121	22	17

Sum	109	354	97	104

**Table 5:** Evaluation results.

Company	Precision	Recall	*F*-measure	Accuracy
CompA	0.47	0.51	0.49	0.66
CompB	0.24	0.34	0.28	0.68
CompC	0.69	0.38	0.49	0.61
CompD	0.85	0.70	0.77	0.69
CompE	0.58	0.64	0.61	0.79

Average	**0.57**	**0.51**	**0.54**	**0.69**
Standard Deviation	0.23	0.16	0.18	0.07
